# *CDK4* Amplification Predicts Recurrence of Well-Differentiated Liposarcoma of the Abdomen

**DOI:** 10.1371/journal.pone.0099452

**Published:** 2014-08-14

**Authors:** Sanghoon Lee, Hyojun Park, Sang Yun Ha, Kwang Yeol Paik, Seung Eun Lee, Jong Man Kim, Jae Berm Park, Choon Hyuck David Kwon, Jae-Won Joh, Yoon-La Choi, Sung Joo Kim

**Affiliations:** 1 Department of Surgery, Samsung Medical Center, Sungkyunkwan University School of Medicine, Seoul, Korea; 2 Department of Pathology, Samsung Medical Center, Sungkyunkwan University School of Medicine, Seoul, Korea; 3 Department of Surgery, The Catholic University of Korea, Yeouido St. Mary’s Hospital, Seoul, Korea; 4 SHIST, Samsung Medical Center, Sungkyunkwan University School of Medicine, Seoul, Korea; The Chinese University of Hong Kong, Hong Kong

## Abstract

**Background:**

The absence of *CDK4* amplification in liposarcomas is associated with favorable prognosis. We aimed to identify the factors associated with tumor recurrence in patients with well-differentiated (WD) and dedifferentiated (DD) liposarcomas.

**Methods:**

From 2000 to 2010, surgical resections for 101 WD and DD liposarcomas were performed. Cases in which complete surgical resections with curative intent were carried out were selected. *MDM2* and *CDK4* gene amplification were analyzed by quantitative real-time polymerase chain reaction (Q-PCR).

**Results:**

There were 31 WD and 17 DD liposarcomas. Locoregional recurrence was observed in 11 WD and 3 DD liposarcomas. WD liposarcomas showed better patient survival compared to DD liposarcomas (*P*<0.05). Q-PCR analysis of the liposarcomas revealed the presence of *CDK4* amplification in 44 cases (91.7%) and *MDM2* amplification in 46 cases (95.8%). WD liposarcomas with recurrence after surgical resection had significantly higher levels of *CDK4* amplification compared to those without recurrence (*P* = 0.041). High level of *CDK4* amplification (cases with *CDK4* amplification higher than the median 7.54) was associated with poor recurrence-free survival compared to low *CDK4* amplification in both univariate (*P* = 0.012) and multivariate analyses (*P* = 0.020).

**Conclusions:**

Level of *CDK4* amplification determined by Q-PCR was associated with the recurrence of WD liposarcomas after surgical resection.

## Introduction

Liposarcomas are the most common type of soft tissue sarcoma accounting for approximately 20% of all soft tissue sarcomas [1], [2]. Well-differentiated (WD) liposarcomas are characterized by atypical but relatively mature adipocyte proliferation, while dedifferentiated (DD) liposarcomas consist of non-lipogenic spindle or pleomorphic cells with elevated mitotic activity [3], [4]. These two histologic subtypes share a common genetic feature characterized by a supernumerary circular and/or giant rod chromosome containing a highly amplified sequence of genes from the 12q13-q21 region [5]. This region contains the *MDM2* and *CDK4*, which are known to play crucial roles in the control of the cell cycle progression [6]. *CDK4* amplification has been observed in several malignancies including glioma, breast cancer, lymphoma, melanoma, and sarcoma [7]. It has been suggested that an absence of *CDK4* amplification in WD and DD liposarcomas is associated with lower rate of recurrence and favorable prognosis [6].

In this study, we sought to identify factors associated with tumor recurrence and patient survival including the levels of *MDM2* and *CDK4* amplification in a homogeneous population of patients with WD and DD liposarcomas of the abdomen undergoing complete surgical resection.

## Methods

### Patient Selection

From December 2000 to December 2010, 139 patients underwent surgical resection for liposarcoma at Samsung Medical Center, Seoul, Korea. Among these patients, 101 cases were diagnosed as WD or DD liposarcomas. Retrospective review was performed for consideration of inclusion in the study. Cases referred to our institute from other centers for management of recurrent tumors were excluded from the analysis. Cases were selected for this analysis when complete surgical resections with curative intent were carried out for WD or DD liposarcomas of the retroperitoneum and peritoneal cavity. Complete surgical resection of the tumor was achieved when all of the following three criteria were met: no gross residual tumor in the surgical field as observed by the surgeon (pathologic R0 or R1 status), histologic confirmation of negative surgical margins and no radiologic signs of residual tumor in the first postoperative follow-up abdomen computed tomography (CT) scan, typically done between postoperative 1 to 4 weeks.

Common practice for surgical resection of abdominal liposarcomas was wide excision of the mass with combined resection of adjacent viscera when the organ is suspected to have direct tumor invasion by preoperative radiologic evaluation and inspection in the surgical field. A tissue expander was left in the space previously occupied by the tumor when adjuvant radiotherapy was planned.

Patients were followed at our outpatient clinic with abdominal CT scans and chest plain x-rays every 3 to 6 months. When local recurrence was suspected, abdominal CT scans were repeated after 1 month or positron emission tomography-CT (PET-CT) was done to confirm the presence of locoregional recurrence or distant metastases. When possible, surgical resection of the recurred tumor mass was attempted. Unresectable tumors or distant metastases to multiple sites were managed with systemic chemotherapy.

This research has been approved by the institutional review board at Samsung Medical Center (Seoul, Korea). All data collection and analysis were done anonymously, and written or verbal consent were not provided by the participants of this work. The lack of consent for this study was also approved.

### MDM2 and CDK4 Quantitative Real-Time PCR

*MDM2* and *CDK4* amplification was analyzed by quantitative real-time polymerase chain reaction (Q-PCR) performed on a PRISM 7500HT Fast Realtime PCR system (Applied Biosystems, Foster City, CA) by using a HotStart-IT SYBR Green qPCR Master mix (USB, Cleveland, OH). Ten nanograms of target DNA was dispensed into each sample with a final reaction volume of 10 µl. Each sample was amplified in triplicate. The PCR was carried out as follows: preheating at 50°C for 2 min and then at 95°C for 10 min, followed by 40 cycles at 95°C for 15 s and 60°C for 1 min. (Primer sequences for each gene are shown in Table 1.) *MDM2* and *CDK4* copy numbers were calculated by comparison to the reference gene (*ALB*) located at 4q11-q13, and were normalized to normal tissue genomic DNA as a calibrator. The level of amplification for *MDM2* or *CDK4* was calculated as follows: copy number of the target gene (*MDM2*, *CDK4*)/copy number of the reference gene (*ALB*). Each gene was considered to have positive amplification when the copy number was greater than 2 times that of the reference gene.

**Table 1:** Primer sequence.

MDM2 (product size = 98 bp)
Forward primer 5′-CCG GAT GAT CGC AGG TG-3′
Reverse primer 5′-AAA AGC TGA GTC AAC CTG CCC-3′
CDK4 (product size = 103 bp)
Forward primer 5′-TTG CAT CGT TCA CCG AGA TC-3′
Reverse primer 5′-CTG GTA GCT GTA GAT TCT GGC CA-3′
Albumin (PCR product size = 81 bp)
Forward primer 5′-TGA AAC ATA CGT TCC CAA AGA GTT T-3′
Reverse primer 5′-CTC TCC TTC TCA GAA AGT GTG CAT AT-3′

bp, base pairs; PCR, polymerase chain reaction.

### CDK4 Immunohistochemical Staining

Immunohistochemical (IHC) staining for CDK4 was performed on representative paraffin-embedded tumor material from the biopsy cut into 4 µm-thick sections and placed onto glass slides. For IHC staining, the slides were deparaffinized in xylene. Staining for CDK4 was performed automatically using the Leica Bond Max immunostainer with Bond Polymer Refine Detection Kits and heat-induced epitope retrieval pH 6.0 (Bond max ER1 (EDTA) solution, Australia) for 15 min. The CDK4 expression was analyzed using mouse monoclonal antibody specific for CDK4 protein (Clone DSC-31, dilution 1∶50, Invitrogen, Carlsbad, CA, USA) [8]. Antibodies against CDK4 react inside the nucleus, so any specific nuclear weak to strong immunostaining was evaluated by microscopy. The cases were stratified into four categories by intensity of the nuclear staining and scores between 0 and 3 were given for each of the categories: negative (0), weak positivity (1), moderate positivity (2), and strong positivity (3). The percentage of tumor cell reactivity was determined, approximately, by visually inspecting the slides and counting 100 tumor cells, at medium/high power, in at least five different fields. The percentage of tumors stained was categorized and scored as follows: 1∼25% (1), 26∼50% (2), 51∼75% (3) and 76∼100% (4). The 2 scores for intensity and percentage given for each case were multiplied to yield the final score of the tumor. A cut-off point for positivity was set at the mean score (6) of the 48 cases analyzed, and a score higher than 6 was considered be indicative of positive expression of CDK4 [9], [10]. Stains were evaluated by two individuals (SYH and YLC) and discordant cases were revised together.

### Statistical Analysis

Chi-square tests (or Fisher’s exact test, when necessary) were used to compare categorical variables. Pearson’s correlation was used to measure the correlation between variables. The Cox proportional hazard model was used for survival and risk factor analysis. All analyses were done using IBM SPSS Statistics version 19 program. Results were considered to be significant when *P*-values were less than 0.05.

## Results

### Patient and Tumor Characteristics

**Figure 1:**
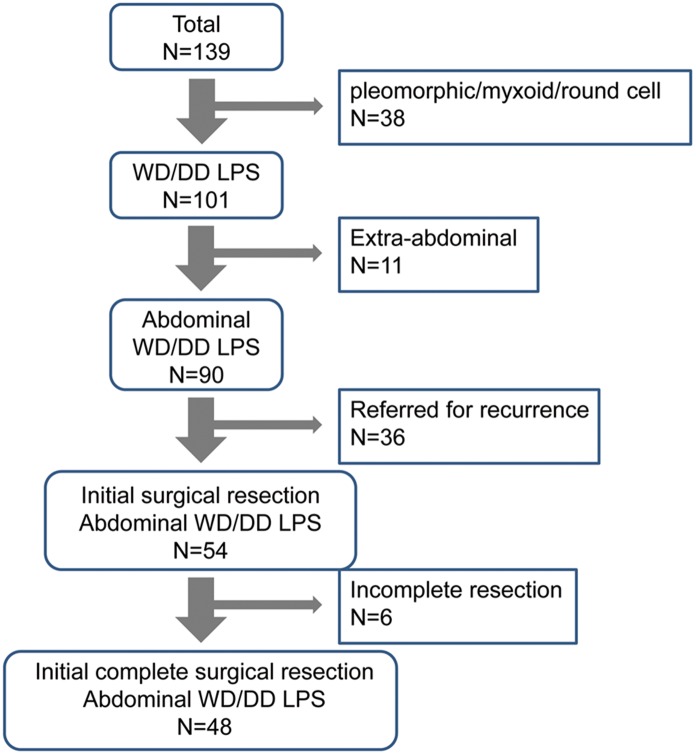
Synopsis of the patient inclusion and exclusion process. (WD, well-differentiated; DD, dedifferentiated; LPS, liposarcoma).

One hundred thirty-nine patients were surgically treated for liposarcoma at our center during the study period. Pathologic examination of the resected tumors of 101 patients showed either WD or DD histologic subtype. Ninety-two tumors were found in the retroperitoneum or peritoneal cavity. Fifty-four patients had their first surgical treatment at our center and 48 had complete surgical resection of the tumor. The patient selection process for inclusion in this study is outlined in Figure 1.

The 48 patients who had complete tumor resection (29 male and 19 female) were included in this analysis. The median age of patients was 57 years (range 37∼78 years). There were 31 WD and 17 DD liposarcomas. Tumors were located in the retroperitoneum in 43 cases, the mesentery in 4 cases and the pelvis in 1 case. Tumor size ranged from 6.8 cm to 60 cm (median 21 cm). The characteristics of the 48 patients and clinicopathological features of the liposarcomas according to histologic type are outlined in Table 2.

**Table 2:** Clinicopathologic characteristics of well-differentiated and dedifferentiated liposarcomas.

	N = 48
Age, median (range)	57 (37∼78)
Sex	
Male	29
Female	19
Histologic type	
Well-differentiated	31
Dedifferentiated	17
Tumor location	
Retroperitoneum	43
Pelvis	1
Mesentery	4
Tumor size, median (range), cm	21.0 (6.8∼60.0)

#### Surgical Treatment and Outcome of WD and DD Liposarcoma

Table 3 shows the surgical and pathological features of the 48 cases according to histologic type of the primary tumor. Combined resection of adjacent viscera was done in 21 of 31 WD and in 15 of 17 DD liposarcomas. An average 1.86 organs were surgically removed during surgery, and the kidney was the organ most often resected with the tumor. Invasion of the resected organ by the tumor was observed upon pathologic evaluation in 7 of 21 WD and 10 of 15 DD liposarcomas (33.3% vs. 66.7%, *p* = 0.021). Microscopic examination of the surgical margin was negative in 11 (35.5%) WD and 3 (17.6%) DD liposarcomas. Tumor recurrence after surgical resection with curative intent was observed in 20 patients (20/48, 41.7%); 11 were WD and 9 were DD liposarcomas (35.5% vs. 52.9%, *p* = 0.144). Recurrence was locoregionally limited in all 11 WD cases while 6 of the 9 DD liposarcomas recurred as distant metastases. A significant difference in recurrence pattern was observed between the 2 histologic subtypes (*p* = 0.001).

**Table 3:** Clinicopathologic characteristics of well-differentiated and dedifferentiated liposarcomas.

	Primary tumor histology	
	Well-differentiated (N = 31)	Dedifferentiated (N = 17)
Extent of surgery		
Mass excision only	10 (32.3)	2 (11.8)
Combined organ resection	21 (67.7)	15 (88.2)
Tumor invasion of resected organ		
Yes*	7 (33.3)	10 (66.7)
No*	14 (66.7)	5 (33.3)
Microscopic resection margin		
Positive	13 (41.9)	11 (64.7)
Negative	11 (35.5)	3 (17.6)
Unknown	7 (22.6)	3 (17.6)
Adjuvant Radiotherapy		
Yes	6 (19.4)	4 (23.5)
No	25 (80.6)	13 (76.5)
Follow-up, median (months)	35.0	18.7
Recurrence		
Yes	11 (35.5)	9 (52.9)
No	20 (64.5)	8 (47.1)
Recurrence pattern		
Locoregional recurrence only**	11 (100)	3 (33.3)
Distant metastasis**	0	6 (66.7)

*p<0.05,

**p<0.01.

**Figure 2:**
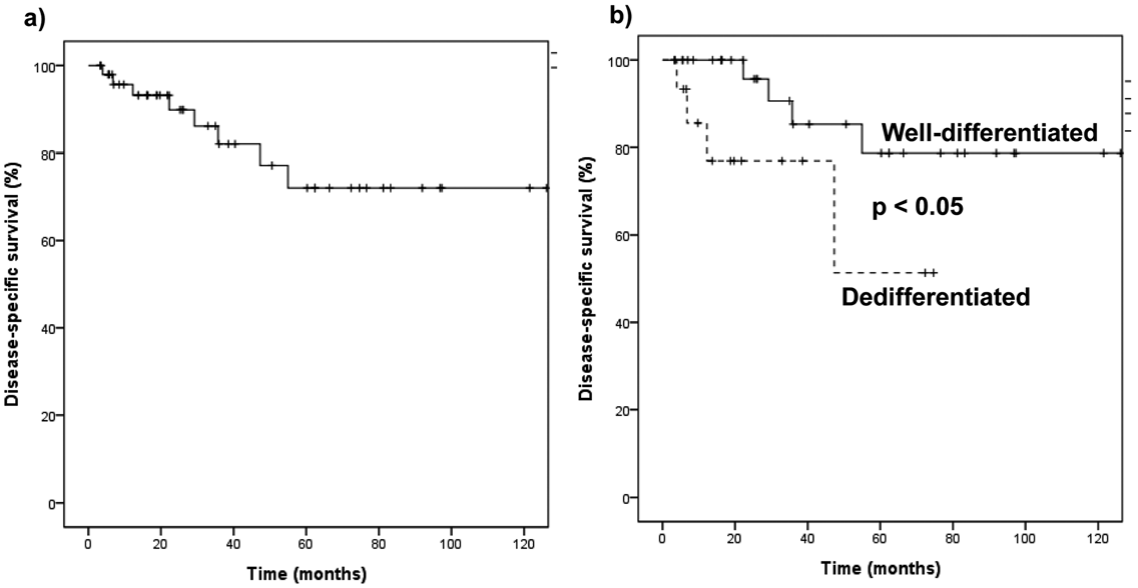
Overall patient survival, a), after surgical resection for well-differentiated and dedifferentiated liposarcoma of the abdomen, and b) Patient survival stratified by histologic subtype of liposarcoma. Well-differentiated liposarcoma group show better survival compared to dedifferentiated liposarcoma group (p<0.05).

Figure 2a shows the disease-specific patient survival of the 48 patients after surgery. Patient survival was approximately 72% at postoperative 10 years. Patient survival according to histologic type is shown in Figure 2b. Kaplan-Meier survival curves showed better patient survival with WD liposarcoma compared to DD liposarcoma (*p*<0.05).

#### Lococregional Recurrence and Distant Metastasis of WD and DD Liposarcoma

Locoregional recurrence was observed in 11 WD and 3 DD liposarcoma cases. The median time interval between surgical resection and the occurrence of the first recurrence was 13.5 months and 11.8 months for WD and DD liposarcomas, respectively. Primary WD liposarcomas expressed variable histologic types in their recurrence lesions; pathologic analyses of the recurrent tumor specimen were WD in 6 cases, DD in 2 cases and pleomorphic in 1 case. All recurrences of primary DD liposarcomas were of the DD histologic subtype. Surgical resection of locoregional recurrence was done in 9 WD and 2 DD cases. Patients underwent an average of 2.0 surgical resections for recurrences (Table 4).

**Table 4:** Locoregional recurrence of abdominal well-differentiated and dedifferentiated liposarcomas.

	Primary tumor histology	
	Well-differentiated (N = 11)	Dedifferentiated (N = 3)
Histologic type of recurred tumor		
Well-differentiated	6	0
Dedifferentiated	2	2
Pleomorphic	2	0
No pathologic evaluation	1	1
Time interval to 1st recurrence, median (range), months	13.5 (4∼120)	11.8 (7∼50)
Treatment of recurrence		
Surgery	4	2
Surgery + radiotherapy	5	0
No Treatment	1	1
Follow-up after recurrence, median (range), months	20.0 (10∼68)	9.6 (4∼22)
Outcome of recurrence		
Dead of disease	5	2
Alive with disease	4	1
No evidence of disease	2	0

Six cases had distant metastases during their follow-up period (Table 5). Of these, the primary tumors were of the DD histologic type in all cases. The median time interval from surgical resection to detection of metastasis was 13.7 months. Metastatic spread was the first evidence of recurrence detected in 4 cases, while in 2 cases, locoregional recurrences were followed by distant metastases. The most common pattern of metastasis was as metastatic nodules in the peritoneal cavity in remote regions away from the primary site (intraperitoneal metastasis) were. One case of liver metastasis and 1 lung metastasis were observed. Metastasic tumor in the deep soft tissue of the thigh was observed in 1 case. Pathologic evaluation of the metastatic lesion was possible in 2 cases and all were DD liposarcomas.

**Table 5:** Distant metastasis of abdominal dedifferentiated liposarcomas.

	N = 6
Site of metastasis	
Intraperitoneal metastasis	4
Liver	1
Lung	1
Thigh	1
Time interval to metastasis, median (months)	13.7
Metastatic tumor histology	
Dedifferentiated	2
No pathologic evaluation	4
Number of recurrences prior to metastasis	
0	4
1	2
Treatment of metastasis	
Surgery	2
Chemotherapy	1
No treatment	3
Median follow-up after metastasis, months (range)	22 (1∼37)
Outcome of metastasis	
Dead of disease	4
Alive with disease	2

Surgical resection of the metastatic tumor was carried out in 2 cases and systemic chemotherapy was done in 1 case. Metastatic liposarcoma was the cause of death in 4 patients. Patient survival after metastasis was 22.2% at 24 months.

### CDK4 and MDM2 Gene Amplification and Immunohistochemistry Results

**Figure 3:**
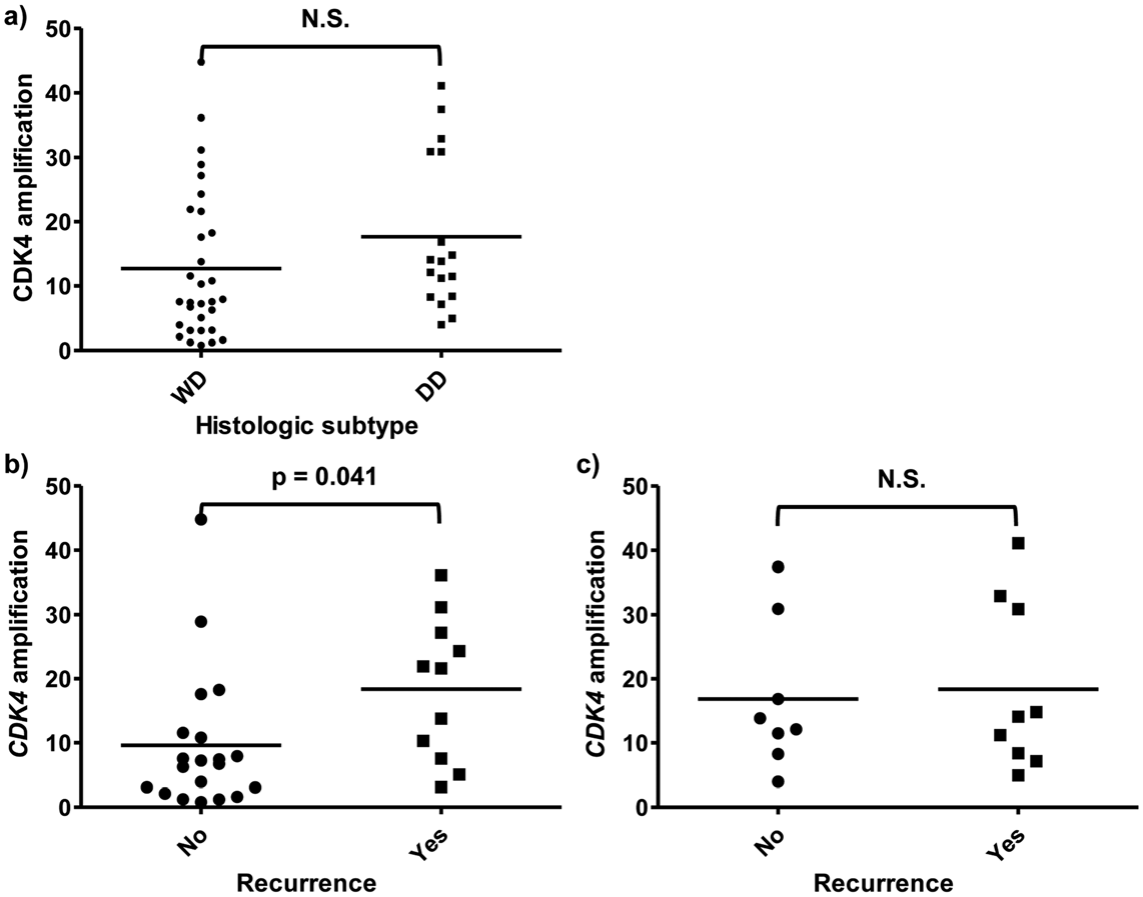
Quantitative real-time PCR results of *CDK4* amplification by a) well-differentiated (WD) and dedifferentiated (DD) liposarcomas: amplification levels were not different between the two histologic subtypes. Quantitative real-time PCR results of *CDK4* amplification in **b)** WD and **c)** DD liposarcomas according to tumor recurrence: amplification was higher in WD liposarcomas with recurrence after surgical resection, but not different in DD liposarcomas regardless of tumor recurrence.

**Figure 4:**
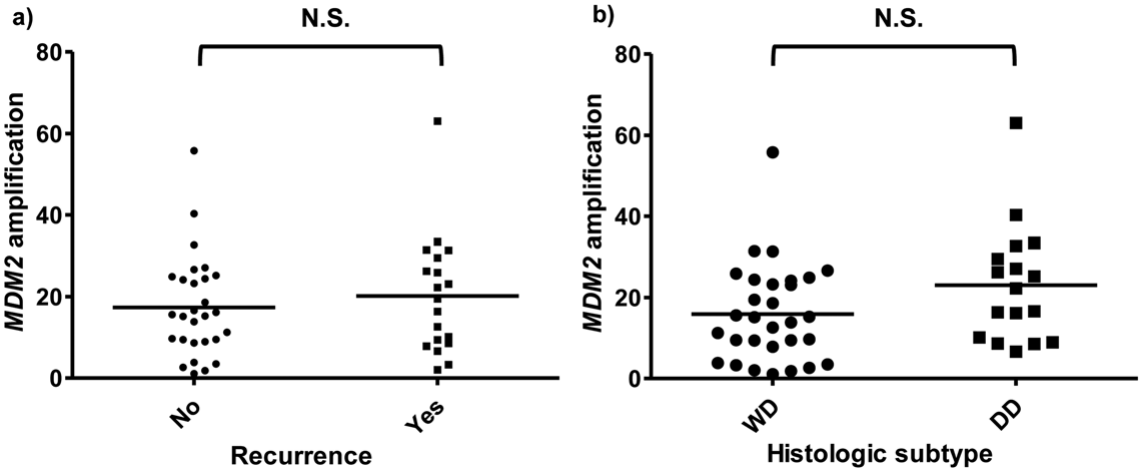
Quantitative real-time PCR results of *MDM2* amplification according to a) tumor recurrence, and b) histologic subtype: *MDM2* amplification levels were not different according to tumor recurrence or histologic subtype.

PCR analysis of the liposarcomas revealed the presence of *CDK4* amplification in 44 cases (91.7%) and *MDM2* amplification in 46 cases (95.8%). The levels of *CDK4* amplification were not different between the two histologic subtypes (Figure 3a). However, among WD liposarcomas, *CDK4* amplification was higher in the subgroup of cases which recurred after surgical resection compared to those without recurrence (*P* = 0.041, independent sample *t*-test, Figure 3b). The levels of *CDK4* amplification were not different in DD liposarcomas, regardless of tumor recurrence status (Figure 3c). *MDM2* amplification levels were not different regardless of histologic subtype or tumor recurrence (Figure 4).

**Figure 5:**
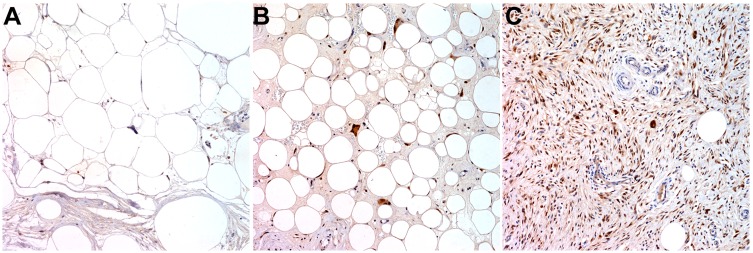
CDK4 immunohistochemistry staining. **A.** CDK4(−) well-differentiated liposarcoma, **B.** CDK4(+) well-differentiated liposarcoma, **C.** CDK4(+) dedifferentiated liposarcoma.

CDK4 immunohistochemistry results were interpretable in all cases. Positive immunoreactivity for CDK4 was observed in 32 out of 48 cases (66.7%, Figure 5). However, positive CDK4 IHC immunoreactivity was not different according to tumor recurrence in all cases (p = 0.363), and in each histological subtypes (p = 0.128 in WD, p = 0.576 in DD; Table 6). The level of *CDK4* amplification by Q-PCR and CDK4 IHC immunoreactivity scores did not show a positive correlation in all cases (*r* = 0.279, *P* = 0.07), and also in each histological subtypes (p = 0.581 in WD, p = 0.112 in DD).

**Table 6:** CDK4 Immunohistochemistry Results According to Tumor Recurrence.

	Overall		WD liposarcoma		DD liposarcoma	
	N = 48	p-value	N = 31	p-value	N = 17	p-value
CDK4 IHC immunopositivity, n (%)						
Recurred	15 (75.0)	0.363	9 (81.8)	0.128	6 (66.7)	0.576
Non-recurred	17 (60.7)		10 (50.0)		7 (87.5)	
CDK4 IHC score, mean ± SD						
Recurred	1.88±2.49	0.745	1.18±2.13	0.758	3.38±2.67	0.251
Non-recurred	1.67±1.41		1.40±1.00		2.00±1.85	

WD, well-differentiated; DD, dedifferentiated; IHC immunohistochemistry.

### Predictors of Recurrence-Free Survival of Liposarcoma

**Figure 6:**
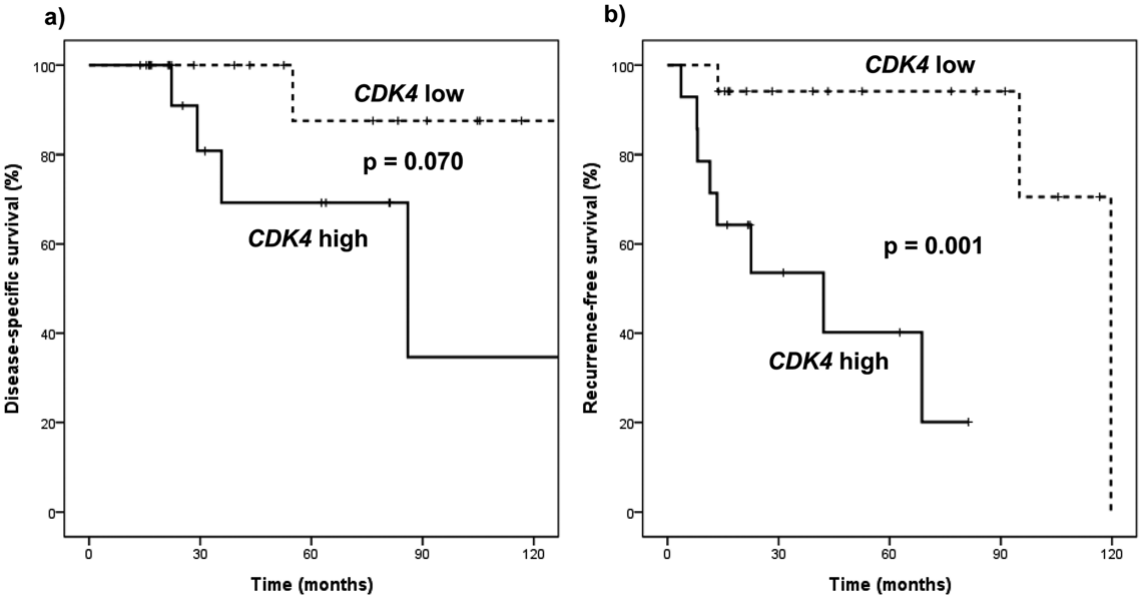
Disease-specific survival, a), and recurrence-free survival of patients, b), with well-differentiated liposarcoma stratified by level of *CDK4* amplification. Low *CDK4* amplification group (*CDK4*≤7.54) showed significantly better recurrence-free survival compared to high *CDK4* amplification group (*CDK4*>7.54).

Predictors of recurrence-free survival of WD liposarcoma are shown in Table 7. High level of *CDK4* amplification (cases with *CDK4* amplification higher than the median 7.54) was associated with poor recurrence-free survival compared to low *CDK4* amplification in both univariate (*P* = 0.012) and multivariate analyses (*P* = 0.020). Disease-specific survival and recurrence-free survival curves of WD liposarcoma patients are shown in Figure 6. Cases with *CDK4* amplification levels greater than 7.54 (*CDK4* high) had significantly inferior recurrence-free survival compared to cases with *CDK4* amplification less than 7.54 (*CDK4* low). None of the analyzed variables were found to significantly affect survival of DD liposarcoma patients.

**Table 7:** Predictors of Recurrence-free Survival for Well-differentiated Liposarcoma in Univariate and Multivariate Analyses.

	Univariate analysis		Multivariate analysis		
	HR	p-value	HR	95% CI	p-value
Older age	1.044	0.122	-	-	-
Tumor size	1.000	0.996	-	-	-
Combined organ resection	1.112	0.872	-	-	-
Adjacent organ involvement	2.912	0.076	-	-	-
Positive resection margin	0.363	0.182	1.491	0.178 ∼ 12.515	0.713
Adjuvant radiotherapy	2.356	0.417	1.681	0.346 ∼ 8.168	0.520
High *MDM2* amplification	22.397	0.638	-	-	-
High *CDK4* amplification	14.608	0.012	12.076	1.476 ∼ 98.833	0.020

HR, hazard ratio; CI, confidence interval.

## Discussion

Amplification of chromosome 12q13-15 is a typical feature of WD and DD liposarcomas [11], [12]. The two main genes in the amplified segment of chromosome 12, *MDM2* and *CDK4*, belong to distinct amplicons and no consistency exists in the amplified sequences between MDM2 and CDK4. It has been well documented that these two genes are involved in the oncogenesis and progression of WD and DD liposarcomas [13], [14]. While *MDM2* amplification and overexpression is present in most WD and DD liposarcomas, *CDK4* amplification is absent in a small proportion of cases [6], [15], [16]. WD and DD liposarcomas with no *CDK4* amplification represent a distinct clinical subgroup with a lower recurrence rate and are more likely to be peripherally located [6]. A limitation of the previously cited study by Italiano *et al.* was the fact that the better prognosis observed in liposarcomas without *CDK4* amplification may have been confounded by these tumors more often being located in the extremities. Liposarcomas of the extremities are more likely to be resected with sufficient free surgical margin compared to those in the retroperitoneum. The liposarcomas included in the current study were limited to tumors arising in the retroperitoneum and peritoneal cavity. Q-PCR of the tumor specimens revealed varying levels of *MDM2* and *CDK4* amplification, with 44 cases (91.7%) and 46 cases (95.8%) showing positive amplification of *CDK4* and *MDM2* genes, respectively. Moreover, WD liposarcomas with recurrence after surgical resection had significantly higher levels of *CDK4* amplification compared to those without recurrence and the degree of *CDK4* amplification was an independent risk factor for recurrence of WD liposarcoma after complete excision. To the best of our knowledge, this study is the first to describe the correlation between *CDK4* amplification and WD liposarcoma recurrence in a quantitative manner. In accordance with the study by Italiano *et al*., four cases in our series that were negative for CDK4 amplification are free of disease recurrence at median 25 months (range 16∼39 months) after surgical resection.

In vitro data on the contribution of *CDK4* to DD liposarcoma progression was presented in a study by Barretina *et al.*, in which shRNA was used to knockdown *CDK4* in two DD liposarcoma cell lines. Sustained knockdown of *CDK4* led to inhibition of proliferation of the cell lines. In addition, pharmacological inhibition of *CDK4* with a selective *CDK4* inhibitor (PD0332991) induced G1 arrest in the same two cell lines [13]. The results of a phase II trial of this drug recently presented show promising prospects for CDK4 inhibition in patients with progressed WD and DD liposarcoma [17]. The results of the current study showed a higher rate of recurrence with increasing levels of amplification of the *CDK4* gene. Our study adds further rationale to the current body of evidence that the use of *CDK4* inhibitors in DD liposarcoma may prove to be beneficial.

Immunohistochemical analysis of CDK4 has been shown to be helpful in the differential diagnosis of liposarcomas and benign lipomatous tumors [9], [18]. In this study, tumors showed positive CDK4 IHC staining in 66.7% of cases. Previous studies have reported positive immunoreactivity for CDK4 on IHC in approximately 68∼70% of liposarcomas [9], [10]. However, IHC results were not associated with histologic subtype of liposarcomas and no significant correlation was seen between CDK4 IHC findings and *CDK4* amplification by Q-PCR. These results suggest that translational or post-translational events function in fine tuning the final outcome of amplication and protein expression can be induced by other mechanism rather than amplication. Discrepancies between CDK4 IHC and Q-PCR findings were also observed in a study by Sirvent *et al.*, in which negative staining by IHC for CDK4 was observed despite the presence of amplification by Q-PCR in approximately 40% of cases of WD and DD liposarcomas [10]. MDM2 IHC analyses were not carried out because we have seen from past reports that *MDM2* was almost ubiquitously overexpressed in WD and DD liposarcomas and therefore did not affect tumor biology [13], [18]. Our *MDM2* Q-PCR analysis results also showed *MDM2* amplification in 46 out of 48 cases in our liposarcoma specimens.

Our group previously analyzed 94 cases of liposarcomas in both the trunk and the extremities and concluded that high grade histologic subtype and positive margin status (microscopic and macroscopic) were independent risk factors for poor survival [19]. In the present study, we have seen consistent results with DD liposarcoma patients showing significantly worse survival compared to WD liposarcoma patients. However, we failed to observe a significant association between surgical margin status and patient outcome. The reason for this may be related to the fact that we have restricted the study population to patients who had achieved complete surgical resection without macroscopically residual tumor, and also the resultant small number of patients analyzed. Previous studies focusing on liposarcomas of the abdomen, particularly the retroperitoneum, emphasized the importance of complete tumor removal during the initial operation [20], [21]. Grossly positive surgical margin was associated with poor disease-specific survival compared to negative surgical margin, while microscopically positive surgical margins did not negatively affect disease-specific survival [21].

In conclusion, the level of *CDK4* amplification determined by Q-PCR was associated with the recurrence of WD liposarcomas of the retroperitoneum and peritoneal cavity. Well-differentiated liposarcomas with higher level of amplification of *CDK4* (≥7.54) were more likely to recur after surgical resection. Utilization of Q-PCR for analysis of *CDK4* amplification may aid clinicians in the postoperative surveillance and management of patients with abdominal WD and DD liposarcomas.
